# Resistance-Nodulation-Division Multidrug Efflux Pumps in Gram-Negative Bacteria: Role in Virulence

**DOI:** 10.3390/antibiotics2010163

**Published:** 2013-03-18

**Authors:** Dinesh M. Fernando, Ayush Kumar

**Affiliations:** Antimicrobial Resistance Research Group (ARRG), Applied Bioscience Program, Faculties of Health Sciences and Science, University of Ontario Institute of Technology, 2000 Simcoe Street N, Oshawa, ON L1H 7K4, Canada; E-Mail: dinesh.fernando@uoit.ca

**Keywords:** multidrug efflux systems, Gram-negative bacteria, host-colonization, oxidative stress, biofilm

## Abstract

Resistance-Nodulation-Division (RND) efflux pumps are one of the most important determinants of multidrug resistance (MDR) in Gram-negative bacteria. With an ever increasing number of Gram-negative clinical isolates exhibiting MDR phenotypes as a result of the activity of RND pumps, it is clear that the design of novel effective clinical strategies against such pathogens must be grounded in a better understanding of these pumps, including their physiological roles. To this end, recent evidence suggests that RND pumps play an important role in the virulence of Gram-negative pathogens. In this review, we discuss the important role RND efflux pumps play in different facets of virulence including colonization, evasion of host defense mechanisms, and biofilm formation. These studies provide key insights that may ultimately be applied towards strategies used in the design of effective therapeutics against MDR Gram negative bacterial pathogens.

## 1. Introduction

Discovery of antibiotics in the early part of the 20th century is considered to be one of the major advances of modern medicine. Our ability to successfully treat bacterial infections has in part led to an unprecedented increase in human life expectancy [1]. However, even around the time antibiotics were first introduced, a number of studies reported resistance to these antibiotics [2]. Nonetheless, since the incidents of resistance were not very widespread and also because newer antibiotics were being discovered quite regularly, antibiotic resistance was not considered a significant problem at the time. This led to the notion that the era of infectious diseases was more or less over [3]. 

Unfortunately, as resistance has continued to spread around the world at a rapid pace, our search for newer antibiotics has become increasingly difficult and has almost come to a standstill in the last few decades. Bacteria utilize various strategies to gain resistance against antibiotics and these mechanisms can be acquired or are intrinsic in nature. Among intrinsic mechanisms of resistance, energy-dependent efflux of antibiotics is considered one of the most important mechanisms of resistance in Gram-negative bacterial pathogens [4,5]. 

Energy-dependent efflux of antibiotics by bacterial cells was first described by Stuart Levy’s group at Tuft’s University, when they showed that tetracycline-resistant strains of *E. coli* were able to pump out the tetracycline molecules thus reducing their effective concentration inside the cell [6]. Efflux of tetracycline may very well have been the underlying mechanism in one of the first reports of tetracycline resistance in the late 1940s [7]. In this classical paper, Demerec showed that tetracycline resistance in *E. coli* was a two-step process, wherein the first step was responsible for low level resistance while the second step conferred high-level resistance. This is consistent with recent findings where efflux has been shown to confer the first but low-level of resistance allowing for the subsequent accumulation of other resistance mechanisms that lead to a higher degree of resistance [8]. 

Efflux proteins identified to date have been classified into five different families: (1) the major facilitator superfamily (MFS); (2) the ATP-binding cassette (ABC) superfamily; (3) the small multidrug resistance (SMR) family; (4) the resistance-nodulation-division (RND) superfamily; and (5) the multidrug and toxic compound extrusion (MATE) family [9]. Of these five families, proteins belonging to the RND family play an important role in the intrinsic resistance of Gram-negative bacteria. Even though these pumps play a critical role in the development of multidrug resistant phenotypes, it is clear that the efflux of antibiotics is not their primary physiological function. An understanding of their natural functions is important in tackling the problem of antibiotic resistance mediated by these pumps, as it will aid in identification of environmental conditions that promote their expression. This knowledge can be used in clinical settings to foresee the emergence of multidrug resistant phenotypes which in turn can help in application of more effective therapeutic interventions. The objective of this review is to underscore the role of RND pumps in the virulence of Gram-negative bacteria with an emphasis on the likely interplay between resistance and virulence.

## 2. Resistance-Nodulation-Division Pumps

RND pumps were first described by two different groups in *E. coli* [10] and *Pseudomonas aeruginosa* [11] independently in the early 1990s. These pumps function as a tripartite complex composed of the RND protein (the inner membrane component), membrane fusion protein (MFP: the periplasmic component), and the outer membrane protein (OMP: the outer membrane protein). These three proteins form a continuous channel across the Gram-negative cell envelope ensuring that the substrate molecule, captured from the outer leaflet of the inner membrane bilayer, is effluxed directly across the periplasm and the outer membrane (which is characterized by low permeability itself) into the external medium with the aid of the proton-gradient as an energy source. In the recent past, crystal structures of the representatives of all three components of the RND complex have been solved [12,13,14,15], providing invaluable insights regarding the mechanism of assembly and action of these pumps. For more information on the mechanism of action of these proteins and their role in multidrug resistance, readers are advised to refer to several excellent reviews published recently [5,16,17]. 

During the initial years after the discovery of RND pumps, it was widely believed that they evolved as a result of antibiotic selection pressure in the bacterial environment. However, it was later shown that RND pumps are part of an ancient family of proteins, homologs of which are found in all three domains of life [18]. This finding led to the belief that since these pumps are omnipresent they must be involved in some important physiological roles. In addition, a single bacterial species can contain multiple RND pumps with overlapping specificities for different antibiotics. For example *P. aeruginosa* contains 12 different RND pumps that share a number of antibiotic substrates. If the primary function of these pumps is to efflux antibiotics, then the redundancy in substrate specificity among different pumps within the same species cannot be easily explained. In addition, antibiotics are not the only known substrates of RND pumps and they are known to efflux a wide range of non-antibiotic compounds including dyes, detergents, disinfectants, fatty acids, *etc.*, further indicating that efflux of antibiotics is not necessarily their primary function. 

The regulation of expression of RND pumps is often mediated by an intricate balance between local regulators (generally repressors encoded upstream of the operon) and global regulators. It is therefore not surprising that the expression of these pumps is very tightly regulated. Overexpression of RND pumps may result from a variety of mechanisms. One mechanism is interaction of selected molecules with the local repressor, thus resulting in derepression of the operon. For example, overexpression of the CmeABC pump of *Campylobacter jejuni* occurs as a result of interaction of its repressor CmeR with salicylate resulting in its inactivation [19]. A second possible mechanism leading to overexpression is mutations in the repressor encoding genes as seen for the overexpression of the MexAB-OprM pump of *P. aeruginosa* or the AcrAB pump of *E. coli* resulting from mutations in their repressor encoding genes *mexR* [20] or *acrR* [21], respectively. Thirdly, activation of expression by means of a global regulator can also lead to pump overexpression. This has been observed with the *E. coli* AcrAB pump which is induced by bile salts and fatty acids as a result of their interaction with the global regulator Rob [22], which is one of the several global activators controlling the expression of this pump.

As a result of their broad substrate specificity, exposure of cells to one substrate that causes overexpression of these pumps can result in cross-resistance to multiple antibiotics. For example, exposure of *P. aeruginosa* cells lacking MexAB-OprM and MexCD-OprJ to triclosan results in isolation of mutants that overexpress the MexJK pump which is also capable of effluxing antibiotics like ciprofloxacin and tetracycline [23]. Various environmental stress conditions, mediated by global regulatory mechanisms, are also known to cause the overexpression of RND pumps and thus contribute to the MDR phenotype. For example ethanol has been shown to cause overexpression of the AdeABC pump of *Acinetobacter baumannii* [24] and the AcrAB pump of *E. coli* [25], incubation temperature has been shown to impact the expression of the EmhABC pump of *Pseudomonas fluorescens* [26] and the AcrAB pump of *Salmonella* Typhimurium [27], and osmolarity has been shown to impact the expression of the AcrAB pump of *E. coli* [28]. 

The extremely broad substrate specificity of RND efflux pumps is also a major hindrance in the discovery and design of new antibiotics, as studies have shown that a majority of promising lead compounds with antibacterial activity can easily be missed in the initial screening process due to their activity [29,30]. In spite of significant advances over the last few years towards a better understanding of the structure and mechanism of the action of RND pumps, the answer to one question remains elusive: what role do they play in bacterial physiology? Studying natural functions of RND pumps poses multiple challenges. For example, broad substrate specificity of these pumps means that not all molecules pumped out are necessarily their ‘natural’ substrate. In addition, although much attention has been paid to their overexpression in response to antibiotics, studies demonstrating their overexpression in the absence of antibiotic selective pressure remain few and far between. 

## 3. Role of RND Pumps in Virulence

For a bacterial pathogen to be able to successfully establish infection in the host, it has to be capable of surviving the host defense mechanisms. Various strategies employed by these pathogens to do so contribute to their virulence. These strategies include overcoming the physical barrier, surviving host antimicrobial components, production of toxins to damage host cells, *etc.* There is now accumulating data that shows that overexpression of efflux pumps in Gram-negative pathogens can occur concomitantly with the process of infection, leading to the question: Is there a correlation between the overexpression of RND pumps and the virulence of a pathogen? In the following sections, we discuss the contribution of RND efflux pumps to various processes contributing to the virulence of bacterial pathogens including; colonization, protection from host defense mechanisms, toxin production, and biofilm production (summarized in Table 1). 

### 3.1. Role of RND Efflux Pumps in Colonization

Several studies have demonstrated the role of RND pumps in host colonization possibly due to their ability to export host-derived antimicrobials as well as bacterial toxins. One of the first such studies was with *P. aeruginosa* which showed that mutants overexpressing the MexAB-OprM, MexCD-OprJ, or MexEF-OprN pumps could be rapidly isolated in the absence of any antibiotic treatment from an acute pneumonia model in rats [31]. Isolation of such mutants in the absence of any antibiotic treatment suggested that overexpression of the RND pumps provided some sort of selective advantage to the bacteria, most likely defense from the host factors. Yet another study showed that the MexAB-OprM pump of *P. aeruginosa* was required for the invasion of Madin-Darby canine kidney (MDCK) cell lines [32]. A third study that supports these findings demonstrated that inhibition of the MexAB-OprM pump resulted in reduced invasion of MDCK cells lines by *P. aeruginosa* [33]. Moreover, MuxABC-OpmB, a recently characterized RND pump in *P. aeruginosa* [34], was shown to contribute to the twitching motility of bacteria [35]. Twitching motility is likely to contribute to the virulence of *P. aeruginosa* as gene deletion mutants of this pump were found to display attenuated virulence in *Brassica pekinensis* and *Drosophila melanogaster* infection models [35]. 

**Table 1:** Examples of Resistance-Nodulation-Division (RND) pumps from Gram-negative species with their antibiotic substrates and proposed physiological role.

Organism	Pump	Antibiotic Substrates	Role in virulence	Reference
*Burkholderia cenocepacia*	BCAL1674-1675-1676	Quinolones	Quorum sensing (autoinducer efflux)	[36]
	BCAL2822-2821-2820	Aminoglycosides, β-lactams, Chloramphenicol, Fluoroquinolones, Quinolones,	Quorum sensing (autoinducer efflux), flagellar motility	[36,37]
	BCAM1945-1946-1947	Aminoglycosides, β-lactams, Ethidium bromide, Fluoroquinolones	Flagellar motility	[37]
*Campylobacter jejuni*	CmeABC	Aminoglycosides, β-lactams, Chloramphenicol, Ethidium bromide Fluoroquinolones, Macrolides, Quinolones, Rifampin, Tetracycline	Host-colonization (bile resistance), Flagellar motility	[38,39]
*Erwinia amylovora*	AcrAB	β-lactams, Ethidium bromide, Quinolones, Tetracycline	Host-colonization (resistance to plant secondary metabolites)	[40]
*Escherichia coli*	AcrAB	β-lactams, Chloramphenicol, Ethidium bromide, Fluoroquinoles, Macrolides, Novobiocin, Rifampin	Host colonization (resistance to bile salts and fatty acids), protection from oxidative damage	[10,28,41,42]
*Neisseria gonorrhoeae*	MtrCDE	β-lactams, Macrolides, Rifampin	Host colonization (resistance to fatty acids, bile salts, steroids, and antimicrobial peptides).	[43,44,45,46]
*Pseudomonas aeruginosa*	MexAB-OprM	β-lactams, Chloramphenicol, Ethidium bromide, Fluoroquinolones,Macrolides, Quinolones, Tetracycline,	Host colonization, invasion of host cells, quorum sensing (efflux of autoinducer)	[11,31,32,33,47,48]
	MexCD-OprJ	β-lactams, Chloramphenicol, Fluoroquinolones, Novobiocin, Tetracycline, Trimethoprim,	Host colonization, biofilm formation (in presence of azithromycin)	[31,49]
	MexEF-OprN	Chloramphenicol, Fluoroquinolones, Trimethoprim	Protection from nitrosative damage, Quorum sensing	[50,51,52]
	MexXY	Aminoglycosides, Macrolides, Tetracyclines	Colonization of cystic fibrosis lung, protection from oxidative damage	[53,54,55]
	MuxBC-OpmB	β-lactams, Macrolides Novobiocin, Tetracycline	Twitching motility	[35]
*Pseudomonas syringae*	MexAB-OprM	Aminoglycosides, β-lactams, Chloramphenicol, Ethidium bromide Fluoroquinolones, Macrolides, Nitrofurontoin, Quinolones, Rifampin, Tetracycline	Host-colonization (resistance to plant secondary metabolites)	[56]
*Salmonella typhimurium*	AcrAB	β-lactams, Chloramphenicol, Fluoroquinolones, Macrolides, Novobiocin, Quinolones,	Host colonization (resistance to bile salts), protection from oxidative damage	[57,58,59,60,61]
*Vibrio cholerae*	VexAB	β-lactams, Macrolides, Novobiocin, Polymyxin B,	Host colonization (resistance to bile salts)	[62]
	VexCD	Novobiocin	Host colonization (resistance to bile salts),	[62]
	VexIJK	β-lactams, Novobiocin,	Host colonization (resistance to bile salts),	[62]
	VexGH	β-lactams, Novobiocin	Host colonization (resistance to bile salts), production of toxins	[63]

In contrast to evidence supporting a role of RND pumps in enhancing virulence of *P. aeruginosa*, there is also evidence to the contrary. For example, one study has shown that overexpression of RND pumps (MexCD-OprJ or MexEF-OprN) can lead to downregulation of type III secretion proteins in *P. aeruginosa* [64]. This in turn is likely to cause a reduction in the virulence of this organism as exhibited by reduced cytotoxicity of pump overexpression mutants on a macrophage cell line. It was also shown in the same study that the overexpression of MexCD-OprJ or MexEF-OprN in clinical isolates of *P. aeruginosa* resulted in a downregulation of *exsA* gene expression [64]. The gene product of *exsA* is a regulator protein that activates the expression of various genes in the type III secretion system regulon [65]. The mechanism by which the overexpression of MexCD-OprJ or MexEF-OprN results in the downregulation of *exsA* gene remains unclear. However, the ExsA protein is known to autoactivate the expression of the *exsA* gene in response to various stimuli [64]. It is therefore possible that overexpression of either of the two RND pumps causes an increased efflux of unknown effector molecule(s) that activate *exsA* expression through the ExsA protein, and this could conceivably cause a reduction in *exsA* expression. Taken together, these studies suggest that different RND pumps may have a different impact on the virulence of *P. aeruginosa*. 

The impact of RND efflux pumps in the pathogenicity of bacteria has also been shown in *Salmonella* Typhimurium. For example, it was found that AcrAB-TolC mutants were attenuated in their ability to colonize mice [59] and chicks [57]. However, the attenuation of colonization was more profound in a TolC deletion mutant than the AcrAB pump, the major RND pump identified in this organism [66]. This is because, while the AcrAB mutant failed to invade macrophages, TolC mutants failed to both adhere to and invade the macrophages [67]. Since TolC is known to function as the outer membrane component of various efflux pumps, it appears that the AcrAB pump may not be the only pump involved in the colonization of *Salmonella* Typhimurium, and that the attenuation of colonization in TolC deletion mutants is a cumulative effect of inhibition of multiple pumps. 

Studies from *Vibrio cholerae* have also provided some interesting insights into the cumulative role of several RND pumps in virulence. It was first shown that four of the six RND pumps present in this organism namely, VexAB, VexCD, VexIJK, and VexGH contributed to the colonization of *V. cholerae* in an infant mouse model [62,63]. Using gene deletions created for the RND protein-encoding genes, the authors of this study showed that the colonization of the small intestines of infant mice was compromised about 50-fold in a strain of *V. cholerae* that lacked all four of the above pumps, while no significant attenuation was observed for various combinations of double knockouts, Δ*vexB* Δ*vexD* [62], Δ*vexB* Δ*vexK* [62], and Δ*vexB* Δ*vexH* [63]. Interestingly, the attenuation of colonization in the strain lacking all three of *vexB, vexD*, and *vexK* genes could be reversed by complementing the strain with the *vexB* gene on a plasmid alone. This presumably results from plasmid-derived overexpression of *vexB* which is likely to be several folds higher than chromosome-based overexpression of the gene. It is clear from these studies that the presence of multiple RND pumps is required for the colonization of the infant mouse intestine by *V. cholerae*. Bile salts are one of the common substrates of all of these pumps and thus it is conceivable that they aid in successful colonization by offering resistance to bile and possibly other antimicrobials present in the host gut. However the purpose of this apparent functional redundancy of RND pumps in the processes of host colonization by *V. cholerae* remains unclear.

In addition to colonization, the same group showed that RND efflux pumps are required for the optimal production of cholera toxin (CT) and the toxin regulated pili (TCP) in *V. cholerae* [62]. It is hypothesized that the VexGH pump in *V. cholerae* is involved in the efflux of effector molecules that suppress the transcription of toxin genes and that their export by RND pumps removes this suppression leading to an increase in expression of CT and TCP [63]. This suggests that RND pumps in *V. cholerae* not only aid in colonization but also in optimum toxin production. 

In addition to the preceding examples of *P. aeruginosa*, *Salmonella* Typhimurium, and *V. cholerae*, RND pumps have also been shown to be important for the colonization of hosts by several other pathogens. For example, the MtrCDE pump of *Neisseria gonorrhoeae* [43] was shown to be critical for the survival of this bacterium in the genito-urinary tract of female mice [44,46]. This environment is rich in fatty acids, bile salt, and steroids, all of which have been shown to be substrates of the MtrCDE pump [44,45]. In addition, this pump has also been shown to impart protection against antimicrobial peptides [68], underscoring the important role the MtrCDE pump plays in resisting the host defense mechanisms and thus aiding in the colonization process. 

The role of RND pumps in colonization has been illustrated in plant pathogens as well. For example, in *Pseudomonas syringae*, a pump similar to MexAB-OprM of *P. aeruginosa* not only effluxes a variety of antimicrobials including dyes, β-lactams, fluoroquinolones, and tetracycline among others, but was also found to play a role in the survival of the organism in bean plants, presumably by protecting cells from plant secondary metabolites [56]. Similarly, the AcrAB pump of *Erwinia amylovora* effluxes secondary plant metabolites with antimicrobial activity, thus aiding in improved colonization of the host [40]. 

### 3.2. RND Efflux Pumps and Phagocytosis

During the process of infection, a pathogen first encounters the elements of innate or non-specific immunity. An important part of the innate immune response is the process of phagocytosis. Here we discuss the correlation between RND pumps and the oxidative and nitrosative stress generated during the process of phagocytosis. Reactive oxygen species (ROS) and reactive nitrogen species (RNS) are important defense mechanisms employed by phagocytic cells against microbial pathogens [69]. As such, pathogenic bacteria must be able to withstand the effects of ROS and RNS in order to survive phagocytosis and cause disease. A number of studies have shown that both oxidative and nitrosative stress can induce the expression of RND pumps suggesting that these pumps are a part of the bacterial defense system against ROS and RNS compounds. 

One of the first suggestions about the link between oxidative stress and RND efflux pumps came from studies in *E. coli* showing that the overexpression of the *soxS* gene can result in upregulation of the AcrAB pump [28,42]. SoxRS is a two-component regulatory system that responds to oxidative stress and therefore plays a role in protection against oxidative damage by the host immune system. SoxR is the sensor protein that activates the expression of *soxS* in response to superoxide and nitric oxide ions. SoxS is subsequently responsible for the activation of genes whose products impart protection against oxidative damage [70]. In *Salmonella* Typhimurium, the SoxRS system has been shown to be involved in the regulation of the AcrAB pump [58]. Indeed clinical isolates of both *E. coli* [70] and *Salmonella* Typhimurium [71] have been isolated that harbor mutations in *soxR* and display the MDR phenotype. The SoxRS system has also been shown to regulate the expression of the AcrAB pump in *Klebsiella pneumonia* [72] and *Enterobacter cloacae* [73] suggesting that the role of oxidative stress in the expression of RND pumps is widespread. In our laboratory, we have also observed upregulation of the AdeABC RND pump in *A. baumannii* in response to oxidative stress (Andrei Bazyleu and Ayush Kumar, unpublished data). Although we are not sure yet if this is a result of the activity of a SoxRS-like system in *A. baumannii*, this does provide further evidence that oxidative stress modulates the expression of RND pumps in bacteria. 

An association between oxidative stress and RND pump expression has also been observed in *P. aeruginosa*. The MexXY pump of *P. aeruginosa* has been shown to be highly expressed in isolates of *P. aeruginosa* from cystic fibrosis (CF) lungs [55] and the expression of this pump has also been shown to be induced by oxidative stress [53]. The MexXY pump is responsible for resistance to aminoglycoside antibiotics [54]. These antibiotics target the ribosome and it is hypothesized that compounds that act on the ribosome are substrates of this pump [74]. Remarkably, the CF lung has been shown to be rich in oxygen radicals [75] and it is therefore likely that an environment rich in oxygen radicals such as CF lungs induces the expression of the MexXY pump which provides a fitness advantage to *P. aeruginosa* in the presence of aminoglycosides [76], the group of antibiotics widely used against this bacterium in CF cases [77]. 

The interplay between oxidative stress and antibiotic resistance is quite interesting as recent studies from the James Collins laboratory at Boston University show that the bactericidal action of a number of unrelated antibiotics results from their ability to generate hydroxyl radicals which ultimately contribute to cell death [41]. The study also revealed that sublethal concentrations of antibiotics induce mutagenesis in *E. coli* by stimulating the production of ROS. One of these mutations was shown to be present in the promoter region of the *acrA* gene that encodes the MFP of the AcrAB efflux pump, thus revealing an intriguing relationship between oxidative stress and RND efflux pumps. If antibiotics elicit their antimicrobial action via generation of ROS, then overexpression of these pumps serves as a useful means to alleviate the bactericidal effects of antibiotics by effluxing these molecules out of the cell.

A recent study in *Campylobacter jejuni* suggests that RND pumps could be part of a complex protection mechanism employed by bacteria against oxidative damage [38]. This study showed that CosR, an OmpR-type response regulator, modulates the expression of almost 500 genes in this organism [78]. Among these are the *cmeABC* operon that encodes an RND efflux pump and the *katA* gene that encodes the sole catalase enzyme in *C. jejuni*. CosR was shown to be a negative regulator of the *cmeABC* operon and a positive regulator of *katA* expression. The CmeABC pump not only effluxes antimicrobials [39] but also plays a critical role in the colonization of chicken intestines, most likely as a result of its ability to efflux bile salts [79]. On the other hand, KatA plays a vital role in resistance to oxidative stress by detoxification of ROS to less toxic compounds. Since efflux pumps have been speculated to play a role in minimizing the effects of oxidative stress [80], possibly by exporting the toxic products of oxidative damage, it can therefore be speculated, based on the counter regulation of *cmeABC* and *katA* by CosR, that the CmeABC pump may be involved in protection against oxidative damage (possibly by effluxing toxic products of oxidative damage) particularly when expression of *katA* is downregulated. 

In addition to ROS, RNS are also an important part of the innate immune system. Just as in oxidative stress, nitrosative stress was also recently shown to induce the expression of the MexEF-OprN pump of *P. aeruginosa* [50], a pump that effluxes a number of antibiotics including chloramphenicol, trimethoprim, and ciprofloxacin [51]. Chloramphenicol however, is the only known antibiotic substrate that is capable of inducing the expression of the *mexEF-oprN* operon [50] whereas a chloramphenicol derivative that lacks the nitro moiety is unable to induce its expression. It is therefore suggested that nitrosated products generated as a result of nitrosative stress are probably natural substrates of the MexEF-OprN pump and that chloramphenicol can induce this pump’s expression as it shares structural similarity with these yet unknown nitrosative products. Homologs of the MexEF-OprN pump have been described in other organisms including the CeoAB-OpcM pump in *Burkholderia cenocepacia* [81], the BpeEF-OprC pump in *Burkholderia pseudomallei* [82], and the AdeFGH pump in *A. baumannii* [83,84]. All of these homologs have been shown to efflux chloramphenicol and the expression of the BpeEF-OprC pump has also been found to be induced by chloramphenicol [85]. However it remains to be seen if chloramphenicol has a similar effect on expression of other homologs of the MexEF-OprN pump. 

### 3.3. RND Efflux Pumps and Biofilms

Biofilms are microbial communities that grow attached to a variety of surfaces. Bacterial biofilms are clinically very important and are implicated in various persistent or recurrent infections that are very difficult to treat with antibiotics. Resistance to various antibiotics in a biofilm results from a multitude of factors that include their impermeability to antibiotics [86], upregulation of various resistance genes [87], and the phenotypic heterogeneity of the cells forming the biofilm [88]. Quorum sensing (QS) is an important mechanism that controls biofilm formation in a cell density-dependent manner [89]. Sensing of the cell density is achieved by means of extracellular signaling molecules called autoinducers (AIs). As the concentration of AIs reaches a certain threshold level, they trigger a signal to bacterial cells which respond by altering the expression of various genes in a coordinated manner. 

The role of RND efflux pumps in biofilm formation has been studied in various organisms. For example, the MexAB-OprM and MexEF-OprN pumps of *P. aeruginosa* have been shown to efflux AI molecules [47,52] effectively reducing their intracellular concentrations. As a result, MexAB-OprM or MexEF-OprN overexpressing strains of *P. aeruginosa* exhibit reduced expression of AI-dependent genes including those involved in AI synthesis itself along with other virulence genes. For the same reason, such strains also exhibit compromised biofilm formation ability. Work from our laboratory also supports this. When comparing two different strains of *P. aeruginosa*, PAO1 (a strain that constitutively expresses the *mexAB-oprM* pump) and PAO200 (a PAO1 derivative lacking *mexAB-oprM*), we observed that even though the secretion of AI, homoserine lactone, was higher in PAO1, PAO200 formed more biofilm than PAO1 (Sarah Warren and Ayush Kumar, unpublished data). Similar observations have been made in *B. cenocepacia* where deletion mutants of two different RND pump-encoding genes *BCAL1675* and *BCAL2821* show approximately 30% less accumulation of AI in the growth medium compared to the wild-type strain [36], further suggesting that RND efflux pumps are involved in the export of quorum sensing signals. 

In addition to a relationship between efflux pump activity and QS, it has also been shown that RND pump expression can impact flagellar motility. Flagellar motility also plays an important role in biofilm formation as it has been shown to impact the entry of macromolecules in the biofilms and also the dissolution of biofilms [90]. Interesting data related to this has come from studies on *B. cenocepacia*. Deletion of an RND pump-encoding gene *BCAL2821* in *B. cenocepacia* was shown to impact the expression of over 200 genes and that of another RND pump encoding gene *BCAM1946* resulted in the altered expression of over 150 genes. In addition, a double deletion mutant of both genes showed differential expression of 550 different genes [37]. A large proportion of genes whose expression was altered in these knock-out strains were those that are involved in flagellar motility. Interestingly, some of the flagellum-associated genes whose expression was upregulated in the *BCAL2821* deletion mutant were found to be downregulated in the *BCAM1946* deletion mutant, leading to speculation that these pumps play a balancing role in flagellum-associated functions. In the same study, the authors showed an increase in biofilm formation in both *BCAL2821* and *BCAM1946* deletion mutants. Since flagellar motility plays a key role in biofilm formation and since the BCAL2821 pump (but not BCAM1946) also effluxes acylhomoserine lactone [36], it is possible that these pumps in *B. cenocepacia* play a role in biofilm formation at multiple levels. For example, they may be involved both in transporting the acylhomoserine lactone and modulating the expression of flagellum-associated genes. 

However, another study in *P. aeruginosa* failed to show any role of the MexAB-OprM, MexCD-OprJ, MexEF-OprN, and MexXY efflux pumps in biofilm formation [91]. A lack of correlation between the expression of RND efflux pumps and autoinducer synthetase genes *lasI* and *rhlI* has also been reported [92]. Nevertheless, in the presence of azithromycin, a macrolide, MexAB-OprM or MexCD-OprJ pumps were found to be required for azithromycin-resistant biofilm formation [93]. These studies show that the role of the MexAB-OprM and MexCD-OprJ pumps in biofilm is complex and dependent on various factors such as the presence of antibiotics. Furthermore, azithromycin has been shown to improve respiratory function in cystic fibrosis patients [94] and the study by Gillis *et al.* [93] shows that the MexAB-OprM and MexCD-OprJ pumps may play a critical role in the formation of biofilms in cystic fibrosis lungs during azithromycin therapy. 

Yet another, albeit indirect, evidence of overexpression of RND efflux pumps in biofilms in *P. aeruginosa* came from the analysis of its multidrug resistance under hypoxic conditions [95]. It was observed that under low oxygen concentrations (1%), *P. aeruginosa* exhibited an increased resistance to various antibiotics. This increased resistance was reversible in the presence of the efflux pump inhibitor (EPI), Phenyl alanine arginine β-naphthylamide (PaβN), suggesting that activity of RND efflux pumps was required for the formation of biofilms. Under hypoxic conditions the expression of MexEF-OprN pumps was found to predominate expression of other RND pumps. Biofilms are structurally very heterogeneous and the center of the microcolonies can have almost no oxygen [87]. It is therefore possible that expression of RND pumps, in this case the MexEF-OprN pump, varies within different layers of biofilms with the highest expression in the deepest layers where the oxygen concentration is the lowest. 

Because biofilms are characterized by the presence of distinct microenvironments, bacterial cells within them can exist in different metabolic states and the outer layers of the biofilm tend to consist of cells that are the most metabolically active. This observation is also relevant with respect to efflux pump activity. For example, the expression of MexAB-OprM has been shown to be dependent on the metabolic state of *P. aeruginosa* with the expression being highest in metabolically active cells [96]. Therefore, cells present in the outer layers of biofilms are more likely to overexpress the MexAB-OprM pump compared to cells present in the inner layers. Though it is not clear whether overexpression of efflux pumps in biofilms serves any other purpose than antibiotic resistance, it is evident that the expression of efflux pumps can vary considerably within different layers of biofilms. 

The role of RND efflux pumps in biofilm formation was also examined in a study where EPIs were shown to significantly reduce biofilm formation in *E. coli* and *K. pneumonia* [97]. The authors of this study used thioridazine (an inhibitor of the NorA major facilitator superfamily efflux pump in *Staphylococcus aureus* [98]) along with two different inhibitors of RND pumps, PAβN and 1-(1-naphthylmethyl) piperazine (NMP). It was observed that the combination of thioridazine with either PAβN or NMP was most effective in inhibiting the formation of biofilms, suggesting that inhibition of multiple efflux pumps belonging to different families may be required for the complete inhibition of biofilm formation. However, since the authors did not explore the effect of EPI combinations on planktonic cells, it is difficult to say if the effect seen was simply the result of deleterious effects on cells from the accumulation of various metabolic byproducts and toxins or if it was specific to biofilm formation. 

## 4. Conclusions

Although RND pump encoding genes have been shown to be present in various bacterial species including pathogenic and non-pathogenic bacteria, the role they play in the multidrug resistance of Gram-negative pathogens makes them extremely clinically relevant. Even though they are arguably the most important contributor to the multidrug resistance phenotype in these pathogens, it is widely accepted that efflux of antibiotics is not the natural function of RND pumps. Study of their natural functions will lead to a better understanding of conditions that promote their expression, information that will be critical in designing improved and more effective therapy options for multidrug resistant organisms. To this end, there is increasing evidence that RND efflux pumps are involved in the virulence of Gram-negative bacteria, thus revealing interplay between resistance and virulence and further highlighting the clinical relevance of these pumps. 

One key implication of these data is that RND efflux pumps are a lucrative target for successful treatment of Gram-negative infections. For example, inhibitors of these pumps can conceivably not only neutralize resistance but also impact their ability to cause successful infection. However, studies on the role of RND pumps in virulence are met with their own challenges. For instance, because of their extremely broad substrate specificity, the mere appearance of a phenotype upon overexpression of these pumps is not enough to ascertain their role in virulence. In addition, functional redundancy of these pumps in colonization or efflux of various ligands (for example, non-antibiotic substrates like quorum sensing molecules and host defense molecules) further complicates efforts to study their contribution to virulence. One approach that promises to provide invaluable information on correlation of antibiotic resistance and virulence mediated by RND pumps is the study of common regulatory mechanisms. For example, identification of common regulatory pathways that control the expression of RND pumps and virulence genes will provide more concrete evidence of their role in virulence, information that can be used to design novel therapeutic options for MDR Gram-negative infections. 
